# Preliminary Study on an Alternative Test Method with MCTT HCE^TM^ for Ocular Irritation Test of Ophthalmic Medical Devices

**DOI:** 10.3390/toxics11030289

**Published:** 2023-03-21

**Authors:** Yang-Jee Kim, Dong-Hyuk Seo, Il-Soo Kim, Mi-Sook Jung, Jin-Young Bae, Moon-Yong Song, Kyung-Seuk Song, Jin-Sik Kim

**Affiliations:** 1 Da Vinci College of General Education, Chung-Ang University, 84 Heukseok-ro, Dongjak-gu, Seoul 06974, Republic of Korea; 2 Korea Conformity Laboratories, 8, Gaetbeol-ro 145 Beon-gil, Yeonsu-gu, Incheon 21999, Republic of Korea; 3 Korea Testing & Research Institute, 12-67, Sandan-gil, Hwasun-eup, Hwasun-gun 58141, Jeollanam-do, Republic of Korea; 4 Biotoxtech, 53, Yeongudanji-ro, Ochang-eup, Cheongwon-gu, Cheongju-si 28115, Chungcheongbuk-do, Republic of Korea; 5 Department of Polymer Science and Engineering, Sungkyunkwan University, 2066, Seobu-ro, Jangan-gu, Suwon-si 16419, Gyeonggo-do, Republic of Korea

**Keywords:** medical devices, eye irritation test, alternative animal test, predictive capacity

## Abstract

The sustained growth of the market for ophthalmic medical devices has increased the demand for alternatives to animal testing for the evaluation of eye irritation. The International Organization for Standardization has acknowledged the need to develop novel in vitro tests to replace animal testing. Here, we evaluated the applicability of an alternative method based on a human corneal model to test the safety of ophthalmic medical devices. 2-Hydroxyethyl methacrylate (HEMA) and Polymethyl methacrylate (PMMA), which are used to fabricate contact lenses, were used as base materials. These materials were blended with eye irritant and non-irritant chemicals specified in the OECD Test Guideline (TG) 492 and Globally Harmonized System (GHS) classification. Then, three GLP-certified laboratories performed three replicates using the developed method using 3D reconstructed human cornea epithelium, MCTT HCE^TM^. OECD TG 492 describes the procedure used to evaluate the eye hazard potential of the test chemical based on its ability to induce cytotoxicity in a reconstructed human cornea-like epithelium (RhCE) tissue. Results: The within-laboratory reproducibility (WLR) and between-laboratory reproducibility (BLR) were both 100%. When a polar extraction solvent was used, the sensitivity, specificity, and accuracy were all 100% in each laboratory. When a non-polar extraction solvent was used, the sensitivity was 80%, the specificity was 100%, and the accuracy was 90%. The proposed method exhibited excellent reproducibility and predictive capacity within and between laboratories. Therefore, the proposed method using the MCTT HCE^TM^ model could be used to evaluate eye irritation caused by ophthalmic medical devices.

## 1. Introduction

The recent growth of the market for ophthalmic medical devices such as contact lenses and intra-ocular lenses (IOLs) [1] has increased the demand for novel methods to evaluate eye irritation. Eye irritation tests are among the three main biocompatibility tests recommended for all medical devices, along with cytotoxicity and sensitization (ISO 10993-10, 2021) [2]. The Draize rabbit eye irritation test, which is based on the OECD Test Guideline 405 [3], is widely used as the international standard (ISO 10993-23) to evaluate whether medical devices cause eye irritation [4]. There are many questions regarding animal testing, such as the validity of the results due to the inherent differences between humans and animals, lack of test reproducibility, and animal welfare issues (e.g., long-term restraint/other types of animal suffering during the testing process). Therefore, the International Organization for Standardization, which establishes international guidelines for the evaluation of medical device biosafety, recommends exploring other in vitro tests to replace animal testing. Particularly, the need for an alternative to animal testing for the evaluation of eye irritation compliant with the ISO 10993-23 guideline has been widely acknowledged.

So far, the most representative test methods based on organ culture models are the bovine corneal opacity permeability test (BCOP, OECD TG437) [5] and the isolated chicken eye test (ICE, OECD TG438) [6], which use the eyes of cattle and chickens after their slaughter. In vitro models based on human corneal tissues have been developed to overcome the drawbacks of organotypic methods that lack comparability with the human eye. The most commonly used reconstructed human cornea-like models (RhCE) are EpiOcular^TM^ (USA) [7], SkinEthic HCE (France), the LabCyte Cornea model (Japan) [8], and MCTT HCE^TM^ (Korea), which are used to test eye irritation for the evaluation of cosmetic and chemical safety (certified as OECD TG 492) [9]. Particularly, the MCTT HCE^TM^ model was developed by isolating human limbal epithelial cells from the corneal tissue remaining after corneal transplant surgery [10,11]. OECD TG 492 describes the procedure used to evaluate the eye hazard potential of the test chemical based on its ability to induce cytotoxicity in a reconstructed human cornea-like epithelium (RhCE) tissue construction [9]. OECD TG 492 recommends incorporations into a testing strategy as an initial step in a bottom-up approach or as the second step in a top-down approach [12]. The methods based on the RhCE models measure cell viability to predict the correct classification of hazardous and non-hazardous substances based on the assumption that all chemicals that cause serious irritation will induce cytotoxic effects in the epithelium. Several studies have sought to evaluate the predictability of eye irritation through a comparative analysis between the existing Draize test method using rabbits and a human corneal model. The RhCE models could distinguish between irritants and non-irritants with high accuracy [13,14,15,16].

Moreover, multi-laboratory verification studies of animal substitution tests for eye irritation have been actively conducted using selected chemicals [17,18]. The predictive capacity and between-laboratory reproducibility of in vitro human corneal assays for the evaluation of eye irritation have been confirmed to sufficiently satisfy the criteria established in the OECD TG 492 performance standards.

However, the aforementioned validation studies were conducted using pure eye irritants and, therefore, did not account for the effects of dilute mixtures such as extracts from polar and non-polar solvents in medical devices [19]. Therefore, additional studies are needed to explore whether the in vitro RhCE models can identify eye irritants in medical device extracts.

Before proceeding to a formal validation study, it is necessary to evaluate the inter-laboratory reproducibility of potential alternative methods and their ability to predict the golden standard. Several in vitro skin irritation tests to evaluate the biological safety of medical devices have been developed using reconstructed human epidermis (OECD TG 439) [20], many of which have been validated and approved under the ISO standard [21,22,23]. However, very few studies have evaluated the applicability of alternative animal tests of eye irritation for ophthalmic medical devices, with a study by Yun et al. [24] being one of the only available examples. Therefore, additional verification studies are urgently needed to evaluate the biological safety of ophthalmic medical devices, including contact lenses.

The present preliminary study for multiple laboratories was conducted to evaluate the accuracy and reproducibility of an alternative eye irritation test method for the evaluation of the biological safety of ophthalmic medical devices using MCTT HCE^TM^ models certified in OECD TG 492.

## 2. Materials and Methods

### 2.1. Preparation of Test Substances

**Figure 1:**
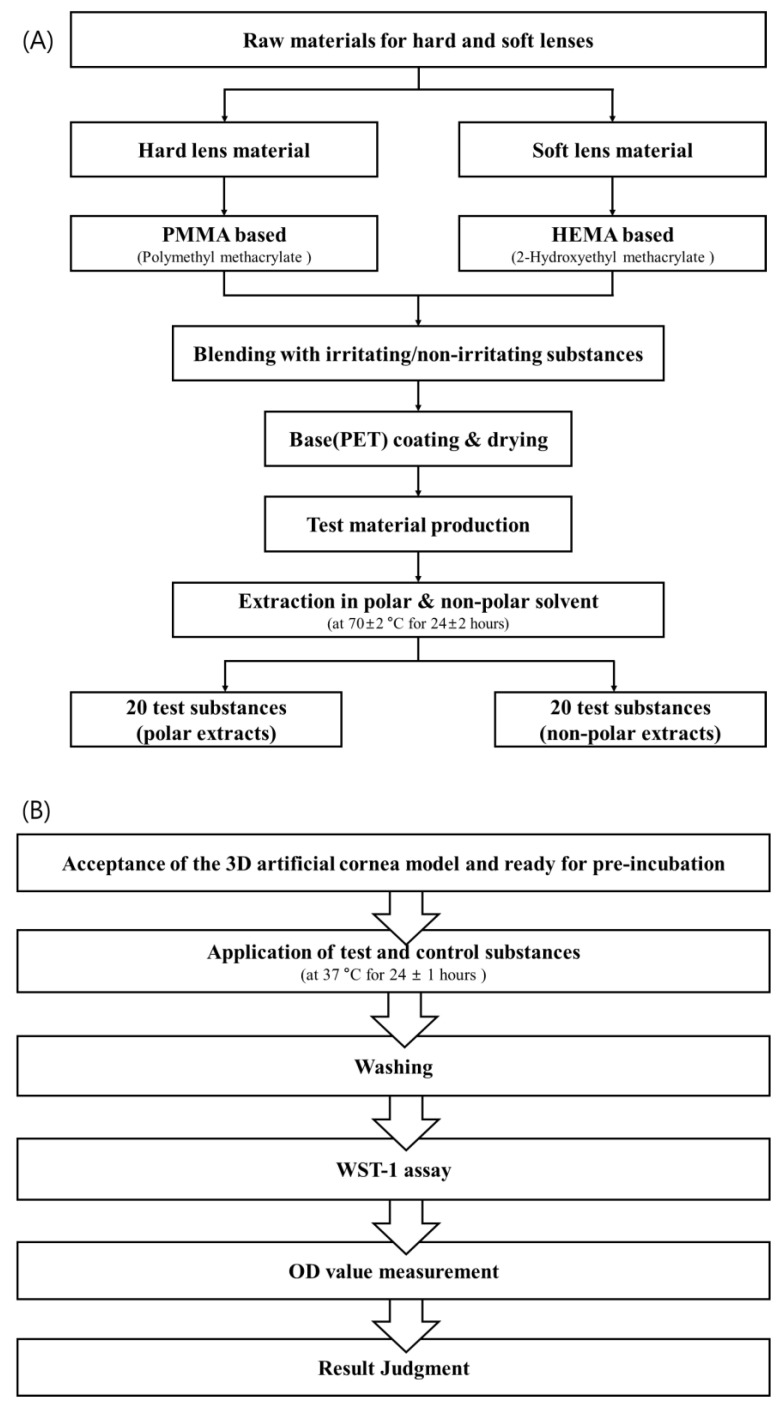
Test substance preparation flow chart and study design. (**A**) Preparation of test substances and (**B**) eye irritation test procedure using MCTT HCE^TM^.

In this study, eye irritation tests for the evaluation of the biological safety of medical devices were performed using medical device extracts as test substances (Figure 1). Polymers are recently being used both as the main materials or coating materials of medical devices. Moreover, the type and amount of substances that can be extracted from medical devices may vary depending on the characteristics of these raw materials. The test materials were prepared by mixing each eye-irritating or non-eye-irritating chemical specified in the OECD Test Guideline (TG) 492 and Globally Harmonized System (GHS) classification into the base material (Table 1).

Polymethyl methacrylate (PMMA, hard lens material) and 2-hydroxyethyl methacrylate copolymer (HEMA, soft lens material) were used as the base materials and mixed with irritating and non-irritating chemicals to produce the testing medical devices as described below (Table 1 and Figure 1).

A blending solution was prepared by mixing 3 g of base material (PMMA or HEMA) with 1 g of an irritant or non-irritating chemical and 17 g of chloroform or tetrahydrofuran (THF) as a solvent. These preparations were then thoroughly mixed using a vortex mixer and a sample shaker (211 rpm, 1 h). The prepared blending solutions were then cast on a PET film using a glass rod to ensure that the film was evenly coated with the solution. Finally, the solvent was removed in a vacuum oven at 50–60 °C for more than 3 h. It was confirmed that the produced testing medical devices were optimally made in this condition. The manufactured testing medical devices were extracted with a polar solvent (sterile physiological saline) and a non-polar solvent (sesame oil) under the following extraction conditions: test substance concentration, 0.2 g/mL; extraction temperature, 70 ± 2 °C; extraction time, 24 ± 2 h [26,27]. The extracts of the medical device are required for a test procedure, and the extraction vehicles and conditions of extraction should be appropriate to the nature and use of the final product and to the purpose of the test. In addition, extraction using both polar (water, physiological saline) and non-polar (freshly refined vegetable oil) extraction vehicles should be performed [26]. The test substances were used within 24 h after the extraction. Using the established test method, 10 irritant and 10 non-irritant extracts were used as the test substances to detect the presence of eye irritants at low levels in medical device extracts.

### 2.2. Participating Laboratories

The three laboratories that participated in the study were the Korea Conformity Laboratories (KCL), the Korea Testing and Research Institute (KTR), and Biotoxtech (BT), all of which were GLP-certified and skilled in the MCTT HCE^TM^ protocol (OECD TG492). All experiments were conducted in a blinded manner. The principal investigator coded and distributed testing medical devices to the experimenters of each laboratory.

### 2.3. Eye Irritation Test Protocol Using MCTT HCE^TM^

Our study used the MCTT HCE^TM^ (Biosolution Co., Seoul, Republic of Korea) 3D artificial cornea model as a test system. Previous studies have described the use of this model to test eye irritation in vitro [10,11]. This study was an eye irritation test using extracts from ophthalmic medical devices and was conducted by directly changing the volume and duration of the treatment from the existing test method using chemicals and cosmetics. It was predicted that a larger amount of test substance and exposure time would be needed than the existing method because it had to be evaluated using the extract. As a result of conducting a pretest by selecting 100 uL and 24 ± 1 h treatment, satisfactory results were obtained. Therefore, the method was adopted for this study (data not shown) and the experimental procedure is described below.

The 3D artificial cornea models were pre-incubated in 900 μL of culture medium (supplied by the manufacturer) in 6-well plates. In preparation for the pre-incubation step, 900 μL of the pre-warmed medium was added to each well of a 6-well plate using a micropipette, and the tissue insert was carefully transferred to the wells using forceps. Then, the well plate was pre-incubated at 37 °C, 5% CO_2_ for 22 ± 2 h. Prior to conducting the test, 100 μL of each positive control, negative control, and test substance were placed at the center of the 3D artificial cornea models. The insert was then held with forceps and turned to ensure that it was evenly coated. After applying the substances, the treated 3D models were incubated for 24 ± 1 h in a 5% CO_2_ incubator at 37 °C. At this time, each substance was used to treat a total of three artificial corneal models. Then, the treated 3D models were washed. To wash the treated 3D artificial corneal model, 4 mL of Dulbecco’s phosphate buffered saline (DPBS; GIBCO, UK) was applied to the inside of the 3D model insert using a pipette aid for each well, overflowing the material inside the insert, and turning the insert using forceps to remove the material inside. Finally, all excess materials were shaken off and washed. Both the inside and outside test substances were removed twice using 10 mL DPBS.

Next, 10 mL of DPBS was taken with a pipette, after which the model was lifted with forceps. The model was then washed twice with DPBS so that both the inside and outside materials were removed. After the washing step, the 3D models were transferred to a 24-well plate containing 200 µL/well of WST-1 (Roche, Basel, Switzerland) solution diluted at a 1:25 ratio, and 100 µL of WST-1 solution was applied to the inside of the 3D model. The 24-well plate containing WST-1 was wrapped in foil to protect the samples from light and incubated for 3 h ± 5 min in a 37 °C, 5% CO_2_ incubator. After incubation, 200 µL of WST-1 formazan per well was transferred to a 96-well plate and the optical density (OD) was measured at a 450 nm wavelength using a spectrophotometer (SpectraMax M2, Molecular Devices, USA).

The tissue viability was calculated using the following equation:

% viability = [(”OD treated tissues - OD blank”)/(”OD negative control - OD blank”)] × 100 (%)

### 2.4. LC-MS/MS Analysis of Extract

Put 1 mL of the extract into a conical tube, add 9 mL of 1 % formic acid (in D.W.) to dilute it, and then add 1 mL of 9 g/L sodium chloride solution to the sample and then Hexane 9 mL, shake at 1200 rpm, and centrifuge at 4000 rpm. Analyze the centrifuged samples as a sample solution using himadzu LCMS 8045 with a Nexera X2 instrument.

### 2.5. Statistical Analysis

#### 2.5.1. Data Collection

Cell viability data for 40 test substances (20 polar extracts and 20 non-polar extracts) were obtained from three replicates performed by each of the three laboratories using the HCTT HCE^TM^ EIT model (i.e., data were obtained from a total of 9 runs per chemical). The three participating laboratories performed the tests and quality assurance following good laboratory practice (GLP) procedures. Table 2 and Table 3 summarize the cell viability data (mean ± SD). The cell viability of the negative control treatment was 100%. A substance is classified as an irritant or non-irritant when cell viability is either below or above 50%, respectively. This criterion applies to both polar and non-polar extracts.

To evaluate the reliability of the test method, the reproducibility within and between laboratories and the capacity of the alternative test method to predict whether a substance is irritant or non-irritant were evaluated as described in the OECD Guidance Document 34 [26]. Statistical analyses were conducted following a previously described protocol to assess reproducibility and predictability [16].

#### 2.5.2. Evaluation of Within-Laboratory and between-Laboratory Reproducibility

Within-laboratory reproducibility (WLR) is an evaluation of the consistency of the results of three replicate tests conducted by a laboratory, and between-laboratory reproducibility (BLR) is an evaluation of the consistency between the three laboratories using the mean value of the results from different independent laboratory tests using a reference substance.

The reproducibility of laboratory results was evaluated based on the proportion of substances showing consistent results for 3 tests out of 20 test materials performed by an institution (Figure 1B). The substances were then labeled as irritant or non-irritant after calculating the average cell viability for each substance based on the 50% cell viability threshold discussed above. All three laboratories must satisfy the reproducibility criteria.

Inter-laboratory reproducibility was determined by calculating the average cell viability for each testing laboratory for 20 test materials performed in each laboratory and determining whether it is an irritant based on the 50% cell viability criterion. This type of reproducibility refers to the proportion of consistent substances in 3 laboratories out of 40 test substances (20 polar extracts and 20 non-polar extracts).

According to the OECD TG 492 guidelines, intra-laboratory and inter-laboratory reproducibility must be at least 90% and 85%, respectively [9]. Wilson’s confidence interval was also calculated to supplement the WLR and BLR estimates. Wilson’s confidence interval is suitable for data analysis with a small number of samples and a binomial distribution. Wilson’s confidence interval was calculated using GraphPad Prism software (Version 8).

Intra-class correlation coefficient (ICC) analysis was used to further evaluate reproducibility. The ICC can determine the reliability between three or more groups and enables the analysis of the consistency of viability between either three laboratories or three experiments in one laboratory. A single measure estimates the extent to which individual measurements from different laboratories or observers vary around their mean value. An average measure, on the other hand, tells us the overall reliability or consistency of the measurements across different laboratories or observers. ICC analyses were conducted using SPSS 26 (IBM SPSS Statistics for Windows).

#### 2.5.3. Evaluation of Predictive Capacity for Each Laboratory

The performance of the MCTT HCE^TM^ model was evaluated by calculating the sensitivity, specificity, and accuracy of the predictive capacity of the outcomes. These metrics provide a basis to measure an assay’s ability to accurately classify the test substances as irritants or non-irritants. These metrics were calculated using the following equations:

Sensitivity (%) = TP/(TP + FN) × 100

Specificity (%) = TN/(TN + FP) × 100

Accuracy (%) = (TP + TN)/(TP + FN + TN + FP) × 100

TP (true positive) is the number of substances correctly identified as irritants; TN (true negative) is the number of substances correctly identified as non-irritants; FP (false positive) is an instance in which a substance is incorrectly identified as an irritant; FN (false negative) is an instance in which a substance is incorrectly classified as a non-irritant. The predictive capacity is expressed as a 2 × 2 contingency table and Wilson’s confidence interval was used to compare the outcomes of the alternative test method with those of the gold standard.

After obtaining the average cell viability of the test results 3 times per laboratory for 40 test substances (20 polar extracts and 20 non-polar extracts), the substances were classified as irritant or non-irritant based on the above-described 50% cell viability criterion. Eye irritation was then determined according to the test results for each laboratory.

## 3. Results and Discussion

### 3.1. Distribution of Overall Data

The 3 repeating test results of 40 test substances (20 polar extracts and 20 non-polar extracts) by different laboratories were expressed in tables (Table 2 and Table 3). 

Our findings indicated that substances could be accurately classified as irritants or non-irritants when a polar extraction solvent was used (Figure S1). However, two irritant substances (PMMA-sodium oxalate and PMMA-sodium dodecyl sulfate) were misclassified as non-irritating when a non-polar extract solvent was used.

To identify the cause of misclassification, we analyzed these extracts using LC-MS/MS. As a result of the analysis, sodium oxalate concentration was 5586 ± 127 ppm and 376 ± 4.4 ppm in HEMA-Sodium oxalate non-polar extract and PMMA-Sodium oxalate non-polar extract (Table 4). Moreover, the Sodium dodecyl sulfate concentration was 27 ± 1.0 ppm and 1.0 ± 0.0 ppm in HEMA-Sodium dodecyl sulfate non-polar extract and PMMA Sodium dodecyl sulfate non-polar extract (Table 4). It was confirmed that these chemicals were better eluted from HEMA than PMMA in the non-polar extract.

### 3.2. Within-Laboratory and between-Laboratory Reproducibility

A fundamental element in the validation of alternative assays is the confirmation of the reproducibility of test results within the same laboratory. Therefore, 3 replicates were performed in 3 laboratories on 40 test substances (20 polar extracts and 20 non-polar extracts) to determine whether the HCTT HCE^TM^ model met the assay criteria specified in the performance standards of the OECD TG 429 guidelines. For this purpose, the test substances used in the multi-laboratory study were tested three times.

#### 3.2.1. WL (Within-Laboratory)/BL (between-Laboratory) Reproducibility Based on Cell Viability

A fundamental element in validating alternative assays is confirming the reproducibility of the test method among the experiments within the same laboratory. Three repeated tests were conducted at each laboratory (Figure 1B). Table 2 and Table 3 summarize the within-laboratory reproducibility (WLR) of the HCTT^TM^ EIT. The WLR for 40 test substances (20 polar extracts and 20 non-polar extracts) is expressed as percentages with Wilson’s score’s confidence interval method to account for the uncertainty of the presented point estimate. All substances were extracted from the polar and non-polar solvents. Data were matched in three repetitions for three laboratories. The WLR was 100% (20/20, 95% Wilson’s confidence interval: 83.9–100) in BT, KTR, and KCL, showing a low variability within each laboratory.

Between-laboratory reproducibility (BLR), which represents the agreement in the performance of a test system between laboratories, was evaluated between the three laboratories based on the average values of the different results. The BLR for both the polar and non-polar solvent extracts was 100% (20/20), meaning that irritants were flawlessly distinguished from the non-irritant substances. These results meet the 90% and 85% criteria for WLR and BLR established in the OECD TG 429 performance standard. However, all three laboratories equally misclassified PMMA-sodium oxalate and PMMA-sodium dodecyl sulfate as non-irritants in non-polar solvent extraction.

#### 3.2.2. Intra-Class Correlation Coefficient Analysis (ICC)

ICC, another primary indicator of an assay’s reliability, was also calculated (Table 5). For each laboratory (BT, KTR, and KCL), the single and average measures were all 0.9 or higher. In both polar and non-polar solvent extraction, the ICC value was 0.9 or higher, indicating high reliability within and between laboratories. ICC is a commonly used indicator to evaluate reproducibility. An ICC value below 0.5 indicates poor reliability, 0.5–0.75 indicates moderate reliability, 0.75–0.9 indicates good reliability, and more than 0.90 indicates excellent reliability [28,29].

### 3.3. Predictive Capacity of the HCTT HCE Model

Both Table 6 and Table 7 show the performance results of the model with all 40 test substances (20 polar extracts and 20 non-polar extracts) in 3 laboratories. The predictive power of the proposed method was evaluated based on its capacity to discriminate irritants from non-irritants according to the average cell viability values determined by each laboratory. All laboratories achieved 100% sensitivity, specificity, and accuracy (Wilson’s CI: 72.3–100) in the polar solvent extraction (Table 6).

Out of the 20 test substances, PMMA-sodium oxalate and PMMA-sodium dodecyl sulfate, both of which are known irritant substances, were misclassified as non-irritants in the non-polar solvent extraction, but the other substances were classified correctly. According to the classification table, the sensitivity, specificity, and accuracy of our proposed approach were 80% (Wilson’s CI: 49.0–94.3), 100% (Wilson’s CI: 72.3–100), and 90% (Wilson’s CI: 69.9–97.2), respectively (Table 7). After integrating the outcomes of the polar and non-polar solvent extract analyses, the overall sensitivity, specificity, and accuracy were all 100% because substances that are classified as an irritant through either extraction approach (polar or non-polar extraction) are ultimately considered an irritant (data not shown). Our data thus demonstrated that our proposed method had a superior predictive capacity compared with that of Yun et al. [24] (sensitivity, 92.6%; specificity, 60.7%; accuracy, 76.7%). Moreover, our approach satisfied all of the criteria for predictive capacity presented in the OECD TG 492 performance standard (sensitivity ≥ 90%; specificity ≥ 60%; accuracy ≥ 75%) (Table 6 and Table 7).

Through various statistical analyses, we found that the reproducibility and predictive capacity within and between laboratories was excellent, demonstrating that the in vitro animal replacement test proposed herein could be used to evaluate eye irritation caused by ophthalmic medical devices.

The irritant substances PMMA-sodium oxalate and PMMA-sodium dodecyl sulfate were likely misclassified as non-irritants when using non-polar solvents. The reason for the difference was confirmed that these chemicals (sodium oxalate and sodium dodecyl sulfate) were less eluted from PMMA than HEMA.

## 4. Conclusions

Our study evaluated an alternative eye irritation test method using the MCTT HCE^TM^ model to evaluate the biological safety of ophthalmic medical devices. The reproducibility and predictability of the proposed eye irritation test method for the evaluation of the biological safety of ophthalmic medical devices were determined using several statistical approaches. The reliability and predictive capacity of the eye irritant tests in medical devices were excellent, achieving an overall accuracy of 100% with a sensitivity of 100% and a specificity of 100%. These results suggest that the proposed eye irritation test method using the MCTT HCE^TM^ model could be applicable for the evaluation of eye irritation for ophthalmic medical devices. Furthermore, the predictive power of both polar and non-polar extraction methods was shown to satisfy the OECD TG 492 PS criteria. Our findings could thus serve as a basis for the incorporation of in vitro alternatives to animal testing for the evaluation of the biosafety of ophthalmic medical devices into an ISO proposal.

## Figures and Tables

**Table 1:** Characteristics of eye-irritating and non-eye-irritating chemicals [3,9,25].

Chemical Name	CAS Number	GHS Category (Eye Irritation)
Hydroxyethyl acrylate	816-61-1	Category 1
Sodium oxalate	62-76-0	Category 1
Sodium dodecyl sulfate	151-21-3	Category 1
Triton X-100	9002-93-1	Category 2A
Benzalkonium chloride	634449-41-2	Category 1
Methylacetate	79-20-9	Category 2A
1-Ethyl-3-methylimidazolium ethyl sulphate	342573-75-5	No category
Dicaprylylether	629-82-3	No category
Piperonyl butoxide	1951-03-06	No category
1-(4-chlorophenyl)-3-(3,4-dichlorophenyl) urea	101-20-2	No category
Physiological saline *	-	No category
Sesame oil *	8008-74-0	No category

* Extraction solvent. All chemicals were purchased from Sigma-Aldrich (Saint Louis, MO, USA).

**Table 2:** Raw data of results of 3 replicates on 20 test substances by each of the 3 laboratories using polar solvent extraction.

										TP	FP	TN	FN
No.	Chemical Name	BT				KTR				KCL			
		1	2	3	Mean ± SD	1	2	3	Mean ± SD	1	2	3	Mean ± SD
1	HEMA-Hydroxyethyl acrylate	0.26	2.73	1.75	1.58 ± 1.24	0.28	0.19	0.12	0.19 ± 0.08	0.32	0.02	0.59	0.31 ± 0.29
2	HEMA-Sodium oxalate	7.87	2.81	27.47	12.71 ± 13.02	3.41	41.48	41.23	28.71 ± 21.91	0.06	0.88	3.31	1.42 ± 1.69
3	HEMA-Sodium dodecyl sulfate	0.69	0.28	0.35	0.44 ± 0.22	0.41	0.25	0.27	0.31 ± 0.09	0.35	0.79	0.61	0.59 ± 0.22
4	HEMA-Triton X-100	0.28	0.87	2.49	1.21 ± 1.14	1.56	0.28	0.19	0.67 ± 0.77	22.57	0.61	2.18	8.45 ± 12.25
5	HEMA-Benzalkoniumchloride	2.05	0.96	3.02	2.01 ± 1.03	0.88	2.01	2.42	1.77 ± 0.80	3.58	6.13	3.53	4.42 ± 1.49
6	PMMA-Hydroxyethyl acrylate	0.67	0.47	1.05	0.73 ± 0.29	0.25	0.16	0.15	0.19 ± 0.05	0.21	0.23	0.49	0.31 ± 0.16
7	PMMA-Sodium oxalate	4.30	21.95	9.66	11.97 ± 9.05	3.62	1.31	2.56	2.50 ± 1.16	0.09	−0.14	2.29	0.75 ± 1.34
8	PMMA-Sodium dodecyl sulfate	8.32	3.13	0.93	4.13 ± 3.79	1.51	0.30	0.31	0.71 ± 0.70	2.44	0.58	1.85	1.62 ± 0.95
9	PMMA-Triton X-100	0.24	0.49	1.12	0.62 ± 0.45	0.26	0.23	0.16	0.22 ± 0.05	0.27	0.58	0.53	0.46 ± 0.16
10	PMMA-Benzalkoniumchloride	2.35	4.64	1.94	2.97 ± 1.46	1.36	0.79	0.92	1.02 ± 0.30	1.42	0.16	0.72	0.77 ± 0.63
11	HEMA	80.46	100.77	97.90	93.04 ± 10.99	114.67	96.18	102.45	104.43 ± 9.40	78.61	56.39	83.37	72.79 ± 14.40
12	HEMA-1-Ethyl-3-methylimidazolium ethyl sulphate	71.54	85.38	72.33	76.42 ± 7.77	80.15	93.85	93.28	89.09 ± 7.75	69.03	65.93	83.18	72.71 ± 9.19
13	HEMA-Dicaprylylether	87.87	94.25	100.72	94.28 ± 6.43	113.78	99.98	91.09	101.61 ± 11.43	95.68	67.32	102.01	88.34 ± 18.47
14	HEMA-Piperonyl butoxide	82.15	87.36	95.43	88.31 ± 6.69	96.23	76.42	96.45	89.70 ± 11.50	84.79	64.25	87.96	79.00 ± 12.87
15	HEMA-1-(4-Chlorophenyl)-3- (3,4-dichlorophenyl) urea	83.29	98.32	96.51	92.71 ± 8.21	105.53	98.89	105.37	103.27 ± 3.79	88.39	68.39	95.82	84.20 ± 14.19
16	PMMA	80.63	83.59	80.94	81.72 ± 1.63	107.76	101.96	101.47	103.73 ± 3.50	93.31	68.93	89.80	84.01 ± 13.18
17	PMMA-1-Ethyl-3-methylimidazolium ethyl sulphate	75.80	99.13	81.26	85.40 ± 12.20	101.35	98.46	92.40	97.40 ± 4.57	85.09	58.64	88.07	77.27 ± 16.20
18	PMMA-Dicaprylylether	75.86	94.71	77.69	82.75 ± 10.40	93.30	95.61	101.69	96.87 ± 4.34	99.30	72.30	94.84	88.81 ± 14.47
19	PMMA-Piperonyl butoxide	76.47	97.05	104.81	92.78 ± 14.64	109.28	105.53	99.86	104.89 ± 4.74	83.82	67.39	99.31	83.51 ± 15.96
20	PMMA-1-(4-Chlorophenyl)-3- (3,4-dichlorophenyl) urea	78.60	78.24	102.99	86.61 ± 14.19	115.07	95.74	92.96	101.26 ± 12.05	91.26	55.72	83.66	76.88 ± 18.72
	Negative control	100.00	100.00	100.00	100.00 ± 0.00	100.00	100.00	100.00	100.00 ± 0.00	100.00	100.00	100.00	100.00 ± 0.00
	Positive control	0.50	0.72	4.49	1.90 ± 2.24	0.74	0.81	6.21	2.59 ± 3.14	10.16	-0.04	0.53	3.55 ± 5.73
Within-Laboratory Reproducibility		100% (20/20)				100% (20/20)				100% (20/20)			
Between-Laboratory Reproducibility		Overall 100% (20/20)											

TP: true positive (Red), TN: true negative (Blue), FP: false positive (Gray), FN: false negative (Yellow). HEMA: 2-Hydroxyethyl methacrylate, PMMA: Polymethyl methacrylate. KCL: Korea Conformity Laboratories, KTR: Korea Testing and Research Institute, BT: Biotoxtech. Negative control: physiological saline. Positive control: methyl acetate.

**Table 3:** Raw data of the results of 3 replicates on 20 test substances by each of the 3 laboratories using non-polar solvent extraction.

										TP	FP	TN	FN
No.	Chemical Name	BT				KTR				KCL			
		1	2	3	Mean ± SD	1	2	3	Mean ± SD	1	2	3	Mean ± SD
1	HEMA-Hydroxyethyl acrylate	5.38	3.77	2.59	3.91 ± 1.40	0.19	0.07	0.45	0.24 ± 0.19	1.16	0.23	0.64	0.67 ± 0.47
2	HEMA-Sodium oxalate	7.38	25.94	37.70	23.67 ± 15.29	33.19	31.15	20.48	28.27 ± 6.83	20.96	21.12	24.12	22.07 ± 1.78
3	HEMA-Sodium dodecyl sulfate	1.35	3.60	6.74	3.89 ± 15.29	0.52	0.30	0.38	0.40 ± 0.11	0.47	0.69	1.22	0.79 ± 0.39
4	HEMA-Triton X-100	0.93	1.74	3.08	1.92 ± 1.09	1.82	1.38	1.20	1.47 ± 0.32	1.47	3.35	3.11	2.64 ± 1.02
5	HEMA-Benzalkoniumchloride	9.88	10.72	2.44	7.68 ± 4.56	0.10	0.70	0.72	0.51 ± 0.35	2.20	0.93	0.62	1.25 ± 0.83
6	PMMA-Hydroxyethyl acrylate	5.89	2.79	7.69	5.46 ± 2.48	0.19	0.23	0.24	0.22 ± 0.03	1.53	1.17	0.25	0.98 ± 0.66
7	PMMA-Sodium oxalate	76.51	86.28	101.45	88.08 ± 12.57	76.47	68.36	65.35	70.06 ± 5.75	97.60	87.28	96.31	93.73 ± 5.62
8	PMMA-Sodium dodecyl sulfate	99.47	99.03	93.79	97.43 ± 3.16	97.76	82.98	110.34	97.02 ± 13.70	87.43	89.09	105.08	93.87 ± 9.75
9	PMMA-Triton X-100	3.20	20.54	2.65	8.80 ± 10.17	3.71	2.18	2.36	2.75 ± 0.84	0.31	2.52	1.67	1.50 ± 1.11
10	PMMA-Benzalkoniumchloride	18.14	10.30	34.62	21.02 ± 12.41	6.18	3.46	11.24	6.96 ± 3.95	1.78	0.50	0.64	0.97 ± 0.70
11	HEMA	77.99	102.02	101.60	93.87 ± 13.75	105.85	90.48	106.19	100.84 ± 8.97	100.72	98.37	115.43	104.84 ± 9.25
12	HEMA-1-Ethyl-3-methylimidazolium ethyl sulphate	96.78	90.08	96.90	94.59 ± 3.90	103.07	90.67	103.58	99.11 ± 7.31	99.44	96.49	116.28	104.07 ± 10.68
13	HEMA-Dicaprylylether	94.23	98.83	101.00	98.02 ± 3.46	108.72	89.83	106.50	101.68 ± 10.33	94.57	95.00	107.87	99.14 ± 7.56
14	HEMA-Piperonyl butoxide	75.07	94.44	88.87	86.13 ± 9.97	102.19	95.40	106.28	101.29 ± 5.50	98.81	99.66	105.22	101.23 ± 3.48
15	HEMA-1-(4-Chlorophenyl)-3-(3,4-dichlorophenyl) urea	86.53	93.35	104.89	94.93 ± 9.28	98.18	93.34	106.57	99.36 ± 6.69	90.07	102.94	81.01	91.34 ± 11.02
16	PMMA	85.09	94.87	114.28	98.08 ± 14.86	111.64	94.75	111.63	106.01 ± 9.75	100.23	97.53	103.98	100.58 ± 3.24
17	PMMA-1-Ethyl-3-methylimidazolium ethyl sulphate	119.19	85.19	99.90	101.43 ± 17.05	104.72	110.26	107.92	107.63 ± 2.79	104.79	104.37	110.49	106.55 ± 3.42
18	PMMA-Dicaprylylether	87.69	101.29	104.83	97.93 ± 9.05	105.77	87.69	99.16	97.54 ± 9.15	95.07	103.77	112.68	103.84 ± 8.80
19	PMMA-Piperonyl butoxide	94.42	88.65	96.64	93.24 ± 4.12	97.50	85.73	105.99	96.41 ± 10.17	100.55	98.64	109.99	103.06 ± 6.08
20	PMMA-1-(4-Chlorophenyl)-3-(3,4-dichlorophenyl) urea	101.84	86.98	107.79	98.87 ± 10.72	96.57	97.51	107.81	100.63 ± 6.23	90.42	102.16	107.32	99.97 ± 8.66
	Negative control	100.54	100.00	100.00	100.18 ± 0.31	100.00	100.00	100.00	100.00 ± 0.00	100.00	100.00	100.00	100.00 ± 0.00
	Positive control	1.67	1.84	4.96	2.82 ± 1.85	0.40	0.49	1.33	0.74 ± 0.52	0.64	1.08	0.46	0.73 ± 0.32
Within-aboratory Reproducibility		100% (20/20)				100% (20/20)				100% (20/20)			
Between-Laboratory Reproducibility		Overall 100% (20/20)											

TP: true positive (Red), TN: true negative (Blue), FP: false positive (Gray), FN: false negative (Yellow). HEMA: 2-Hydroxyethyl methacrylate, PMMA: Polymethyl methacrylate. KCL: Korea Conformity Laboratories, KTR: Korea Testing and Research Institute, BT: Biotoxtech. Negative control: sesame oil. Positive control: methyl acetate.

**Table 4:** Chemical analysis data using LC-MS/MS.

Chemical Name	Analysis Chemical	Concentration (ppm)
HEMA-Sodium oxalate	Sodium oxalate	5586 ± 127
PMMA-Sodium oxalate	Sodium oxalate	376 ± 4.4
HEMA-Sodium dodecyl sulfate	Sodium dodecyl sulfate	27 ± 1.0
PMMA-Sodium dodecyl sulfate	Sodium dodecyl sulfate	1.0 ± 0.0

**Table 5:** Intra-class correlation coefficient (ICC) for the test results within and between laboratories using polar and non-polar solvent extraction.

	Laboratory		ICC (CI) Polar Solvent Extraction	ICC (CI) Non-Polar Solvent Extraction
WLR	BT	Single measures	0.966 (0.922–0.986)	0.953 (0.900–0.980)
		Average measures	0.989 (0.972–0.995)	0.984 (0.964–0.993)
	KTR	Single measures	0.977 (0.953–0.990)	0.980 (0.944–0.992)
		Average measures	0.992 (0.984–0.997)	0.993 (0.981–0.997)
	KCL	Single measures	0.931 (0.780–0.975)	0.985 (0.963–0.994)
		Average measures	0.976 (0.914–0.992)	0.995 (0.987–0.998)
BLR	Overall	Single measures	0.967 (0.903–0.988)	0.988 (0.974–0.995)
		Average measures	0.989 (0.966–0.996)	0.996 (0.991 -0.998)

KCL: Korea Conformity Laboratories, KTR: Korea Testing and Research Institute, BT: Biotoxtech.CI, 95% confidence interval.

**Table 6:** Predictive capacity of the test results of three repetitions in three laboratories using polar solvent extraction.

Reference Result	Test Result (BT)		Test Result (KTR)		Test Result (KCL)		Total	
	I	NI	I	NI	I	NI	I	NI
I	10	0	10	0	10	0	10	0
NI	0	10	0	10	0	10	0	10
Total	10	10	10	10	10	10	10	10
Sensitivity (%)	100		100		100		100	
Specificity (%)	100		100		100		100	
Accuracy (%)	100		100		100		100	

I, irritant: % viability ≥ 50%. NI, non-irritant: % viability < 50%. KCL: Korea Conformity Laboratories, KTR: Korea Testing and Research Institute, BT: Biotoxtech.

**Table 7:** Predictive capacity of the test results of three repetitions in three laboratories using non-polar solvent extraction.

Reference Result	Test Result (BT)		Test Result (KTR)		Test Result (KCL)		Total	
	I	NI	I	NI	I	NI	I	NI
I	8	2	8	2	8	2	8	2
NI	0	10	0	10	0	10	0	10
Total	8	12	8	12	8	12	8	12
Sensitivity (%)	80		80		80		80	
Specificity (%)	100		100		100		100	
Accuracy (%)	90		90		90		90	

I, irritant: % viability ≥ 50%. NI, non-irritant: % viability < 50%. KCL: Korea Conformity Laboratories, KTR: Korea Testing and Research Institute, BT: Biotoxtech.

## Data Availability

The data presented in this study are available upon request from the corresponding author.

## Supplementary Materials

The following supporting information can be downloaded at: https://www.mdpi.com/article/10.3390/toxics11030289/s1, Figure S1: Scatter plot results of three replicates on test substances by three laboratories. The first 10 of 20 substances are irritants, and the latter half are non-irritants. The pink lines indicate the mean ± SD of each laboratory (error bar) from three replicates. The chemical numbers correspond to the chemicals listed in Table 2 and Table 3.
