# Supplementary material for: Preliminary Study on an Alternative Test Method with MCTT HCETM for Ocular Irritation Test of Ophthalmic Medical Devices
Source: Toxics. 2023 Mar 21;11(3):289. doi: 10.3390/toxics11030289 (PMC10051360; doi:10.3390/toxics11030289)
Supplement: Supplementary file 1 [file toxics-11-00289-s001.zip › toxics-2260730-SI.pdf]

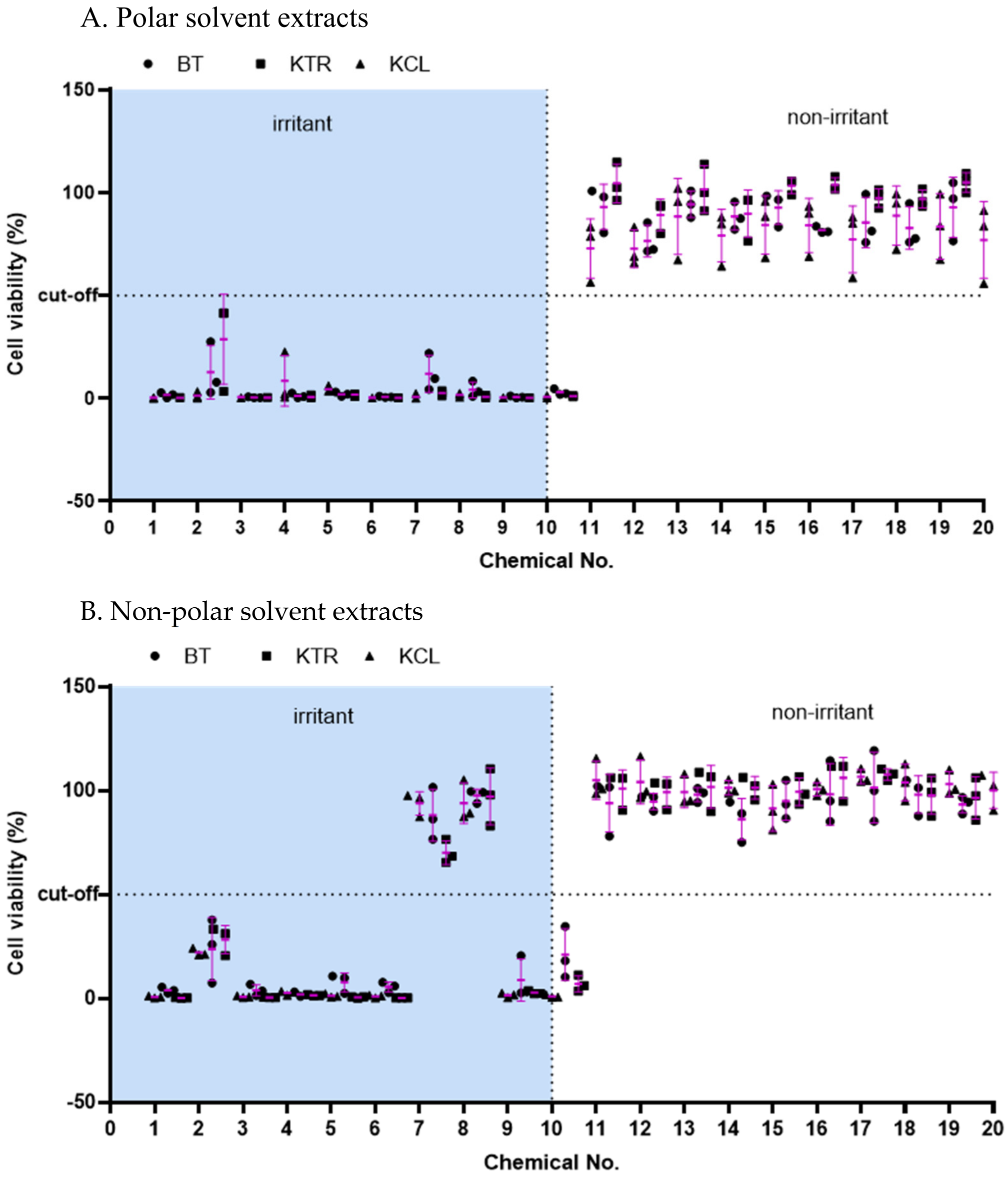

Figure S1. Scatter plot results of three replicates on test substances by three laboratories. The first 10 of 20 substances are irritants, and the latter half are non-irritants. The pink lines indicate the mean ± SD of each laboratory (error bar) from three replicates. The chemical numbers correspond to the chemicals listed in Tables 2 and 3. .
